# P22 Paraneoplastic diffuse systemic sclerosis in association with ano-rectal tumour

**DOI:** 10.1093/rap/rkac067.022

**Published:** 2022-09-28

**Authors:** Katarzyna Nowak, Gary Wright

**Affiliations:** Musgrave Park Hospital, Belfast, United Kingdom; Musgrave Park Hospital, Belfast, United Kingdom

## Abstract

**Introduction/Background:**

Rheumatic diseases can manifest as a paraneoplastic phenomenon. They can be seen during the course of neoplastic disease, precede the diagnosis, appear simultaneously or occur after the diagnosis of malignancy. Overall, paraneoplastic rheumatic syndromes are rare. It is however important to be aware of this phenomenon as the signs and symptoms of the rheumatic syndrome will usually subside with treatment of the underlying malignant disease. Furthermore, reappearance of symptoms may suggest cancer relapse.

**Description/Method:**

51 year old woman with no significant past medical history was referred by her General Practicioner with 6-week history of pain and stiffness in hands, arms and legs. There was also associated 4-week history of change in bowel habit (diarrhoea and faecal incontinence). Clinical examination revealed skin thickening proximal to elbows and knees, sclerodactyly, facial telangiectasia as well as tendon friction rubs in lower limbs. There was also a mass palpable on digital rectal examination. Investigations revealed that the patient had new iron deficiency anaemia and mildly raised carcinoembryonic antigen. Anti-RNA polymerase III antibody returned positive. CT of chest, abdomen and pelvis suggested tumour involving the anus and lower rectum with metastatic lymphadenopathy. Those findings were also confirmed on subsequent MRI pelvis and PET CT. She underwent anal biopsy, examination under anaesthesia and formation of loop colostomy. Histopathology results confirmed poorly differentiated squamous carcinoma. Patient was then reviewed by oncology and discussed at multidisciplinary meeting. She was commenced on palliative chemotherapy (paclitaxel and carboplatin). After the first session she became severely anaemic and developed acute renal failure. She passed away approximately three months after initial referral to rheumatology.

**Discussion/Results:**

This patient was diagnosed with paraneoplastic diffuse systemic sclerosis in association with ano-rectal tumour. Unfortunately at the time of diagnosis the disease was metastatic therefore the only treatment option available was palliative rather than curative. Paraneoplastic rheumatic syndromes are rare. The signs and symptoms are secondary to the presence of malignancy. They may be induced by the substances that are secreted by the tumour or the immune system’s response to the malignant cells. Usually once patient recovers from the neoplastic disease the rheumatic disease signs/symptoms subside. In our patient’s history there were a number of features which raised the possibility of paraneoplastic aetiology. These included: sudden onset and rapid progression of clinical symptoms, late onset of disease (after 50 years of age), family history of cancer (dad had prostate cancer), new iron deficiency anaemia as well as the presence of anti-RNA polymerase III antibodies. This antibody is linked with cancer-associated systemic sclerosis. Patients who are positive have shorter cancer-scleroderma interval. Anti-RNA polymerase III antibody is unique to scleroderma and is not found in cancer patients without scleroderma.

**Key learning points/Conclusion:**

The key learning point from this case was not to forget the paraneoplastic aetiology in patients who present with rheumatic syndromes. In particular if the symptoms are of sudden onset or are progressing rapidly/responding poorly to therapy. There is only a small number of cases reported in literature of paraneoplastic systemic sclerosis. Furthermore, the importance of anti-RNA polymerase III antibody testing in scleroderma patients. If positive, it is important to be aware that those patients have >4-fold increased risk of cancer occurring within 2 years of scleroderma onset. It is therefore vital to look for any signs/symptoms during their routine follow-up appointments at rheumatology clinics.

